# Novel Highly Divergent SARS-CoV-2 Lineage With the Spike Substitutions L249S and E484K

**DOI:** 10.3389/fmed.2021.697605

**Published:** 2021-06-28

**Authors:** Katherine Laiton-Donato, Jose A. Usme-Ciro, Carlos Franco-Muñoz, Diego A. Álvarez-Díaz, Hector Alejandro Ruiz-Moreno, Jhonnatan Reales-González, Diego Andrés Prada, Sheryll Corchuelo, Maria T. Herrera-Sepúlveda, Julian Naizaque, Gerardo Santamaría, Magdalena Wiesner, Diana Marcela Walteros, Martha Lucia Ospina Martínez, Marcela Mercado-Reyes

**Affiliations:** ^1^Dirección de Investigación en Salud Pública, Instituto Nacional de Salud, Bogotá, Colombia; ^2^Centro de Investigación en Salud para el Trópico - CIST, Facultad de Medicina, Universidad Cooperativa de Colombia, Santa Marta, Colombia

**Keywords:** SARS-CoV-2, lineage, COVID-19, spike, variant of interest

## Abstract

COVID-19 pandemics has led to genetic diversification of SARS-CoV-2 and the appearance of variants with potential impact in transmissibility and viral escape from acquired immunity. We report a new and highly divergent lineage containing 21 distinctive mutations (10 non-synonymous, eight synonymous, and three substitutions in non-coding regions). The amino acid changes L249S and E484K located at the CTD and RBD of the Spike protein could be of special interest due to their potential biological role in the virus-host relationship. Further studies are required for monitoring the epidemiologic impact of this new lineage.

## Introduction

COVID-19 continues challenging the health system abroad. After the emergence of SARS-CoV-2 in China in late 2019 and despite the rapid international response once the WHO declared it as a Public Health Emergency of International Concern (PHEIC), the virus rapidly crossed the borders, started autochthonous transmission in every country and spread locally despite the strict lockdown measures (1). The enormous population size of SARS-CoV-2 at the global level and its RNA nature has led to the rapid accumulation of genetic variability as more than 800 lineages (2, 3). Some lineages or genetic variants have attracted special attention from the beginning of the pandemic spread to date (4, 5), due to their rapid increase in frequency in some areas, abnormally high mutation accumulation across the genome, most amino acid changes affecting the spike protein, evidence for evolutionary convergence of some critical changes and increasing evidence for virus escape to the antibody-mediated immunity (6–9). As genomic information is being deposited in public databases, a growing number of lineages or variants of interest (VOI) and concern (VOC) is being reported (https://github.com/cov-lineages/pango-designation/issues). Interestingly, a very high and increasing number of lineages containing the E484K substitution in the Spike protein have been reported to emerge independently at least 67 times and worldwide (Table 1). This amino acid change located at the RBD of the spike protein has been found to have a negative effect on neutralization by monoclonal antibodies (10), as well as vaccine-induced (11) and polyclonal antibodies resulting from natural infection with circulating lineages (12).

**Table 1:** List of lineages and date of emergence of the E484K substitution in the Spike protein^a^.

**Lineage + 484K**	**Country of first reported sequence**	**Earliest collection date**	**Lineage + 484K**	**Country of first reported sequence**	**Earliest collection date**
A	Nigeria	2021-01-07	B.1.146	Brazil	2021-01-11
A.2	Spain	2020-03-31	B.1.160	Netherlands	2020-11-07
A.23.1	England	2020-12-26	B.1.165	England	2021-01-08
B.1	Switzerland	2020-03-21	B.1.177	Switzerland	2020-09-30
B.1.1	USA	2020-12-09	B.1.177.4	England	2020-11-14
B.1.1.1	England	2020-06-06	B.1.2	Canada	2020-12-24
B.1.1.10	England	2020-12-26	B.1.214	Belgium	2021-01-28
B.1.1.103	Bangladesh	2020-12-19	B.1.234	USA	2021-01-14
B.1.1.105	USA	2021-01-04	B.1.235	Nigeria	2021-01-08
B.1.1.130	Russia	2020-11-17	B.1.237	South Africa	2020-09-16
B.1.1.145	South Africa	2020-10-09	B.1.241	USA	2020-07-08
B.1.1.163	Israel	2020-12-24	B.1.243	USA	2021-01-18
B.1.1.164	Australia	2020-04-16	B.1.260	USA	2021-01-06
B.1.1.184	Russia	2020-11-09	B.1.274	Kuwait	2020-12-27
B.1.1.207	USA	2020-11-18	B.1.279	Canada	2020-04-20
B.1.1.220	USA	2020-12-31	B.1.280	USA	2021-01-14
B.1.1.235	England	2020-08-17	B.1.281	Bahrain	2020-11-09
B.1.1.269	Belgium	2020-11-10	B.1.311	USA	2021-01-15
B.1.1.273	South Africa	2020-10-05	B.1.316	England	2020-11-14
B.1.1.28	England	2020-12-08	B.1.351	South Africa	2020-10-08
B.1.1.29	USA	2020-12-28	B.1.369	USA	2020-10-06
B.1.1.305	USA	2021-02-04	B.1.400	USA	2020-12-10
B.1.1.306	India	2020-09-01	B.1.416	Gambia	2020-09-22
B.1.1.316	Bangladesh	2021-01-19	B.1.441	Egypt	2021-01-03
B.1.1.33	Brazil	2020-11-11	B.1.486	USA	2020-10-22
B.1.1.34	South Africa	2020-10-06	B.1.487	USA	2021-01-15
B.1.1.57	South Africa	2020-10-09	B.1.525	England	2020-12-15
B.1.1.67	Singapore	2021-01-22	B.1.526	USA	2020-12-16
B.1.1.7	England	2020-12-17	C.1	South Africa	2020-11-10
B.1.1.74	England	2020-04-30	D.2	Australia	2020-10-27
B.1.1.85	USA	2021-01-08	H.1	South Africa	2020-12-21
B.1.110.3	USA	2020-09-01	P.1	Brazil	2020-12-04
B.1.134	USA	2020-08-25	P.2	Brazil	2020-04-15
B.1.139	USA	2020-05-22			

a *Analysis performed for the sequences available up to February 14, 2021 in GISAID*.

In Colombia, SARS-CoV-2 genomic surveillance was established early during the pandemic, leading to the identification of the importation of at least 12 lineages before international flight cancellation and during lockdown (13). A high percentage (48%) of SARS-CoV-2 sequences were assigned to the B.1 parental lineage with little or no shared mutations accumulated during the early local transmission inside the country. Thereafter, the microevolution of the virus allowed the emergence of some lineages, including the B.1.111 and B.1.420, which were considered Colombian lineages, due to a major representation of sequences from Colombia (37.4 and 85.4%, respectively) in GISAID by February 28, 2021.

Here we report a novel and highly divergent lineage with 21 characteristic mutations, including 10 non-synonymous, eight synonymous and three mutations in non-coding regions (5'and 3' UTR and intergenic region). Further studies are required to assess the functional role of these mutations and to monitor their epidemiologic impact.

## Methods

### Genomic Surveillance

Genomic surveillance was established at the Sequencing and Genomics Group, National Institute of Health, Colombia (http://www.ins.gov.co/Noticias/Paginas/coronavirus-genoma.aspx). Samples for Next Generation Sequencing (NGS) were selected from routine surveillance in all departments and special groups based on clinical and epidemiologic criteria (14). A total of 287 complete genomes were processed during the period from March 2020 to February 2021. Processing of RNA samples was performed as previously described (13), with the implementation of suggested modifications to the amplicon sequencing protocol (Arctic LoCost) (15) and NGS raw data processing following the protocol described for ONT (https://artic.network/ncov-2019/ncov2019-bioinformatics-sop.html). A dataset including Colombian sequences of SARS-CoV-2 representative of the different lineages and those previously reported in GISAID (Supplementary Table 3) with substitutions of special interest was prepared and used for recombination detection through the RDP4 software (*P*-values < 0.05) (16), adaptive evolution analysis at the codon level through IFEL and MEME (*P*-value < 0.3) (17) and phylogenetic analysis.

### Lineage Assignment and Phylogenetic Reconstruction

Lineage assignment was performed through the Pangolin algorithm 2 (github.com/cov-lineages/pangolin). *p*-distance-was calculated for intra-lineage and between-lineages at nucleotide level. Maximum likelihood phylogenetic reconstruction was performed with GTR+F+I nucleotide substitution model using IQTREE (18). Branch support was estimated with an SH-like approximate likelihood ratio test (SH-aLRT) (19).

## Results

Four sequences from samples collected in Colombia between December 26, 2020 and January 14, 2021 presented a characteristic mutation pattern, including two amino acid changes in the Spike protein (L249S and E484K). These sequences were originally assigned to the B.1.111 lineage by Pangolin COVID-19 Lineage Assigner (https://pangolin.cog-uk.io/) and currently reassigned to the B.1 lineage (Pango Lineage version: 2021-04-01). The lineage B.1 has been the major basal and widespread lineage from the initial SARS-CoV-2 spread and it became the more prevalent lineage in Colombia (13), while the B.1.111 lineage, first detected in the USA from a sample collected on March 7, 2020 and subsequently in Colombia on March 13, 2020 is currently circulating and mainly represented by Colombian sequences from all around the country (https://microreact.org/project/vHdc5J3MeoYJ2u69PLP6NF).

**Figure 1:**
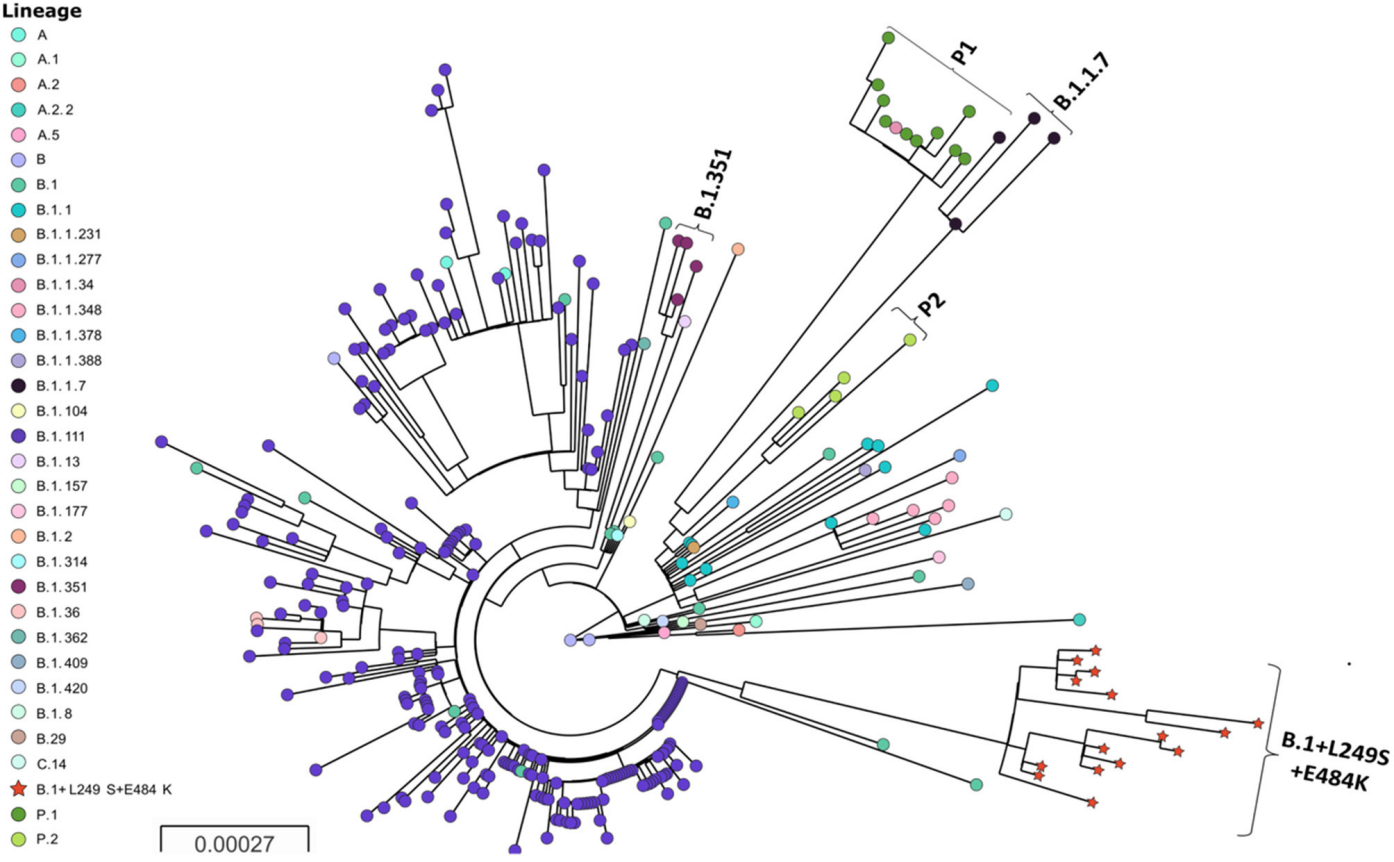
Phylogenetic tree of SARS-CoV-2 and B.1+L249S+E484K emergence. Major lineages circulating in Colombia and representative sequences of the VOC are depicted. The tree was reconstructed by maximum likelihood with the estimated GTR+F+I nucleotide substitution model for the dataset of 304 full-length genomes. The interactive tree can be accessed in the following link: https://microreact.org/project/fTa6f3kY9JraG9NPmQYGog/42c3e045. Red stars represent the sequences belonging to the new lineage.

The phylogenetic analysis allowed to identify a highly distant lineage clustering the sequences containing the+L249S and E484K amino acid changes (Figure 1). The inclusion of SARS-CoV-2 sequences representative from the different lineages circulating in Colombia, as well as sequences representative of the major lineages and VOC circulating worldwide allowed to demonstrate the emergence of a novel and phylogenetically distant lineage of SARS-CoV-2 (provisionally named: B.1+L249S+E484K). While it has been detected in several countries, the phylogenetic relationship and the earliest collection date of a sequence belonging to this lineage suggest a recent emergence in ColombiaB.1 was shown to be the more recent common ancestor and therefore the parental lineage, while B.1.111 continues being closely related at the national level. No putative recombination events were detected for the analyzed dataset (data not shown).

The large list of distinctive mutations at the nucleotide and protein levels (Table 2) are consistent with the existence of a common recent ancestor for the Colombian sequences and other reported sequences from USA (eight sequences), Aruba (two sequences) and Belgium (one sequence). The B.1+L249S+E484K intra-lineage (0.000208 substitutions per site between each pair of sequences) and between-lineages *p*-distances (0.000733–0.001918 substitutions per site between each pair of sequences) suggest a drastic divergence of the new lineage from the most closely related lineages (Supplementary Table 2). While increasing the sample size could help to reconstruct the gradual accumulation of mutations leading to divergence from the B.1 ancestor, a plausible explanation for the origin of this highly distant lineage could be the existence of a strong selection pressure on the virus population in an unknown context (e.g., natural infection in a population reaching herd immunity, convalescent plasma or monoclonal antibodies treatment, chronic infection in immunocompromised patients, replication in a different vertebrate species, etc.) (10, 20–22). The result of the analysis by IFEL and MEME, despite the low significance, is suggestive of the presence of a weak but positive selection signal in seven codons, including the previously identified position 614 in the Spike protein (Supplementary Table 3) (23).

**Table 2:** Nucleotide and amino acid substitution pattern of the B.1+L249S+E484K putative lineage.

**Genomic coordinates**	**B.1++L249S+E484K putative lineage substitutions**	
	**Amino acid change**	**Region/Protein**
C241T	NA	5'UTR
C1060T	Synonymous	NSP2
T5213C	Synonymous	NSP3
C5221T	Synonymous	NSP3
G10523C	V3420L	NSP5 (3C-like proteinase)
C16694T	T1076I	NSP13 (Helicase)
G17211T	L1248F	NSP13 (Helicase)
G17721T	V2365F	NSP13 (Helicase)
T21294A	Synonymous	NSP16 (2'-O-ribose methyltransferase)
G21295A	Synonymous	NSP16 (2'-O-ribose methyltransferase)
G21296A	G2610N	NSP16 (2'-O-ribose methyltransferase)
T22308C	L249S	Spike
G23012A	E484K	Spike
C26681T	Synonymous	Membrane glycoprotein
A27253G	I18V	ORF6 protein (Nonstructural protein NS6)
C27442T	Synonymous	ORF7a protein (Nonstructural protein NS7a)
C27741T	Synonymous	ORF7a protein (Nonstructural protein NS7a)
G27798T	A15S	ORF7b protein (Nonstructural protein NS7b)
A28272T	NA	Intergenic
C28887T	T205I	Nucleocapsid phosphoprotein
G29781T	NA	3'UTR

## Discussion

Genomic surveillance in real time is critical for the identification of genetic changes that could be potentially associated to the epidemiologic and clinical behavior during COVID-19 pandemic. Several VOC and VOI are being described from the end of 2020 to date. VOC are characterized by very high number of mutations located at the Spike protein, whose evidence of biological significance started to accumulate. In the present study, the emerging lineage is bearing the amino acid change E484K, located at the receptor binding domain (RBD) of the Spike protein. This change is of special relevance as it has been associated to the phenotypic properties of some well-described VOC and several VOI (4, 7–9). E484K has been suggested to be responsible for a considerably lower neutralizing activity *in vitro* from convalescent plasma (20, 24, 25), although the cell-mediated immunity could not be affected by the distinctive mutations (26). In the same way, despite it has not been considered a critical amino acid change, S249L is located at the N-terminal domain (NTD), the second domain most frequently targeted by neutralizing antibodies (25). The potential impact of E484K in concert with other amino acid chances has been suggested for the P.1 variant (8), therefore, its effect in combination to S249L or other changes in critical proteins for viral replication (e.g., Helicase, 2'-O-ribose methyltransferase, etc.) found in the here reported lineage is to be determined.

Despite increasing effort in the routine genomic surveillance in Colombia, the new lineage has only been detected from samples collected during late December to mid-January mainly from the Caribbean region of the country, which supposes a major effort is necessary to determine the epidemiologic contribution and potential expansion in the different cities.

An obligatory question that arises from the current analysis of the novel lineage and the evidence of 67 other lineages with the evolutionary convergence at the Spike E484K is related to the context of the emergence of highly divergent lineages, and the selection of specific substitutions. The fact that some amino acid changes have appeared independently in these lineages is not plausibly explained by chance, but probably by the result of a selective immune pressure. Many hypotheses have been raised without conclusive support. One of them is related to the chronic infection in immunocompromised patients and the administration of under-neutralizing antibody titers during convalescent plasma or monoclonal antibody therapies (21, 22, 27, 28) also raising questions about the use of immunotherapies for treatment of acutely infected patients.

In the context of pandemic spread of the virus, an enormous virus population size is expected, as it is also the emergence of virus variants that could also make possible the emergence of antibody-resistant mutants in the context of natural infection in immunocompetent people. Therefore, another plausible hypothesis for the emergence of neutralization escape mutants could be the fact that several countries and cities approximated to a high seroprevalence during the second semester of 2020 and became more restrictive for transmission of the first wave lineages, privileging the growth of specific lineages with distinctive mutations that allowed the escape to the polyclonal immune response.

It is mandatory to evaluate the impact of the genetic background of B.1+L249S+E484K in the neutralization efficacy of convalescent sera/plasma from acquired immunity.

## Data Availability Statement

The datasets presented in this study can be found in online repositories. The names of the repository/repositories and accession number(s) can be found below: https://www.gisaid.org/, Full genome SARS-CoV-2 Colombian sequences belonging to the new proposed lineage were deposited in GISAID under accession numbers: EPI_ISL_1092008, EPI_ISL_1092007, EPI_ISL_1092006, and EPI_ISL_1092005.

## Ethics Statement

The studies involving human participants were reviewed and approved by Comité de Ética y Metodologías de la Investigación–CEMIN, Instituto Nacional de Salud, Bogota, Colombia. Written informed consent for participation was not required for this study in accordance with the national legislation and the institutional requirements.

## Author Contributions

KL-D and MM-R designed the study and planned the experiments. KL-D, CF-M, DA-D, HR-M, JR-G, DP, SC, MH-S, JN, GS, and MW carried out the experiments. DW, MO, and MM-R supported the epidemiologic aspects of the study. KL-D and JU-C analyzed the data and took the lead in writing the manuscript. All authors reviewed the final manuscript, contributed to the article, and approved the submitted version.

## Conflict of Interest

The authors declare that the research was conducted in the absence of any commercial or financial relationships that could be construed as a potential conflict of interest.
